# Acute kinematic changes following low-intensity tethered swimming as a constraints-led priming strategy in competitive swimmers

**DOI:** 10.1186/s13102-026-01896-2

**Published:** 2026-07-27

**Authors:** Maciej Skorulski, Szymon Kuliś, Magdalena Olszewska, Jan Gajewski

**Affiliations:** 1 Józef Piłsudski University of Physical Education in Warsaw, Warsaw, Poland; 2 Doctoral School, Józef Piłsudski University of Physical Education in Warsaw, Warsaw, Poland

**Keywords:** Front crawl, Tethered swimming, Motor priming, Stroke length

## Abstract

**Background:**

Traditional pre-activation protocols, such as post-activation performance enhancement, often rely on high-intensity stimuli, which may induce peripheral fatigue and impair fine motor control. This may be particularly problematic in swimming, where technical precision is critical. Therefore, this study examined the acute effects of low-intensity technical tethered swimming (LITTS) as a constraints-led pre-activation strategy.

**Methods:**

Thirty-two adolescent competitive swimmers (mean age 13.2 ± 0.6 years) completed two pre-activation conditions on separate testing days: LITTS and a non-tethered swimming condition. Approximately 90 s following the intervention (for equipment setup), participants performed a maximal 50-m front-crawl trial. Acute technical changes were assessed using triaxial accelerometry-derived kinematic variables. Statistical comparisons were performed between conditions and pre-post responses.

**Results:**

Following LITTS, significant post-intervention changes in swimming kinematics were observed. In particular, stroke length significantly improved following LITTS (F(1,29) = 47.09, *p* = 0.001, η^2^ = 0.61). In addition, 50-m performance time was significantly shorter following LITTS compared with the free-swimming condition (F(1,29) = 9.71, *p* = 0.005, η^2^ = 0.24).

**Conclusions:**

LITTS may be an effective low-fatigue proprioceptive priming strategy in adolescent swimmers. The findings suggest that this approach may acutely improve selected swimming kinematics and short-distance swimming performance, offering a technically oriented alternative to traditional high-intensity priming protocols.

**Trial registration:**

ClinicalTrials.gov, NCT07554248. Registered 17.04.2026 (retrospectively registered). https://clinicaltrials.gov/study/NCT07554248

## Introduction

The difference between a medal-winning time and that of a non-finalist in the 50 m freestyle at the Olympic Games is minimal. For example, at the Paris 2024 Olympic Games, the gap was only 0.13 s in the men’s event and 0.27 s in the women’s event [1]. Accordingly, it is unsurprising that both researchers and swimming coaches continually seek ways to improve performance through technical refinement. Swimming technique influences performance primarily by reducing hydrodynamic drag and optimising propulsive force application, both of which directly affect race times [2, 3].

Warming up is widely recognised as an essential component of preparation for physical activity. Its importance has been extensively documented in the literature [4–7]. It provides measurable benefits in swimming, with reviews and meta-analyses indicating improved sprint performance. Although the literature on warm-ups in swimming is extensive, including recent reviews, the main emphasis has been placed on thermodynamic and metabolic changes in muscles, while detailed biomechanical analysis has been overlooked [7, 8].

Priming is a phenomenon whereby prior exposure to a stimulus before a task temporarily calibrates the nervous or muscular system [9]. The aim is to enhance the effectiveness of the target task. Priming has been more extensively studied in the context of post-activation potentiation (PAP) [10], and post-activation performance enhancement (PAPE) [11]. Most existing research focuses on the physiological mechanisms of potentiation (PAP/PAPE). PAP and PAPE represent physiological subtypes of priming, both relying on high-intensity stimuli to enhance subsequent performance [12, 13]. However, in technically complex cyclical sports such as swimming, increasing power output alone is not sufficient to maximise performance. Hydrodynamic resistance increases non-linearly with velocity, meaning that even small improvements in movement pattern efficiency – through better force application or reduced drag – may yield greater performance gains than increases in physical capacity alone [14].

Tethered swimming is widely described in the literature as a training modality [15–18]. While fully tethered protocols have been widely used for force and technique assessment, semi-tethered modalities, using elastic cords in resistive or assistive modes, have also been employed as training tools [19]. Tethering is primarily used as a high-intensity training modality. However, recent research has expanded the range of proposed applications, suggesting its use as a low-intensity training method aimed at improving swimming technique [20]. It is worth noting that technical tethered swimming (TTS) may serve as an aquatic equivalent of constraints-led technical priming strategies used in other sports [21], although it operates via a different kinematic mechanism [20].

From the perspective of the constraints-led approach (CLA) to movement instruction, the environment and task are modified so that the desired kinematic changes emerge through the interaction between the athlete, the environment, and the task (self-organisation). In this sense, TTS is not an external load, but a task constraint that forces the athlete's nervous system to seek the most effective movement solution [22]. Within this theoretical framework, the proposed mechanism is as follows: in free swimming, forward velocity provides a stabilising framework for the moving body. In tethered swimming, however, the average velocity is effectively zero (no net forward displacement), so this effect disappears. This suggests that improper force application may induce sudden rotational perturbations that are absent during free swimming – a phenomenon referred to here as hydrodynamic instability.

This type of hydrodynamic instability is consistent with the principle of error augmentation, a movement-teaching strategy in which task constraints amplify movement errors, thereby increasing their salience and potentially eliciting stronger corrective responses [23]. In the present context, tethering may serve as such a constraint, though the corrective mechanisms were not directly assessed.

Previous studies have demonstrated that low-intensity technical swimming exercises may acutely influence stroke kinematics and neuromuscular readiness prior to performance [20, 24]. However, to the best of our knowledge, no previous study has examined whether low-intensity technical tethered swimming (LITTS) can function as a constraints-led technical priming strategy in competitive swimmers. The available literature therefore provides limited information on the acute effects of tethered technical exercises on stroke kinematics, and further research is warranted.

Recent research conducted by Kotuła et al. [21] demonstrated the existence of technical priming in hurdling. The study evaluated how three different priming strategies affect mechanical variables and sprint kinematics. One strategy involved technical wicket drills, in which five 40-m accelerations were performed through a series of mini-hurdle ‘wickets’ positioned at equal intervals and individually adjusted to each athlete’s stride length, in order to reinforce the stride rhythm and geometry imposed by the hurdle configuration. Of the three priming strategies, interlimb asymmetry and mediolateral sway exerted the strongest effects. In this approach, improvements in performance were not attributable to increased strength or power, but rather to the reinforcement of ideal rhythmic work and movement regulation through sensory-motor calibration [25]. However, it is not possible to transfer the wicket drills model directly to swimming, mainly due to the lack of hard ground.

The aim of this study was to examine the acute effects of LITTS on selected kinematic variables in competitive swimmers, a pattern of change we tentatively refer to as acute technical refinement (ATR). ATR is operationally defined here as a pattern of improvement across selected kinematic variables assessed immediately after the intervention, without a concomitant increase in physiological load.

It was hypothesised that, following LITTS exposure, participants would show acute improvements in free-swimming kinematics. A possible mechanism is hydrodynamic instability induced by tethering, which may promote sensorimotor adaptation during subsequent free swimming, although this mechanism was not directly assessed in the present study.

## Materials and methods

The study included 15 girls (age 13.18 ± 0.66 years; body height 165.86 ± 5.97 cm; body mass 52.47 ± 4.76 kg; body fat 20.37 ± 2.88%) and 17 boys (age 13.29 ± 0.63 years; body height 168.61 ± 9.14 cm; body mass 53.96 ± 9.53 kg; body fat 14.1 ± 2.3%). Body weight and composition were assessed at baseline, prior to the first testing session, using a Tanita BC-545N scale (Tanita Corporation, Tokyo, Japan). All participants had been involved in regular swimming training since approximately 9–10 years of age. They were competitive swimmers from a local sports club, training 7–8 times per week and regularly competing in national competitions. According to the Participant Classification Framework proposed by McKay et al. [26], participants were classified as Tier 3: Highly Trained/National Level, based on their participation in national-level competitions. Performance-based criteria were adapted for the junior athlete context, as recommended by the authors of the framework. Exclusion criteria included injury, failure to meet the age requirements, or insufficient training experience, defined as less than 2 years of regular training experience.

Half of the participants had already trained using tethered swimming before the study commenced. The remainder, who had not previously trained with tethered swimming, completed a 15-min verbal and observational familiarisation session prior to testing, during which the procedure, execution, common difficulties, and key focus points were explained and demonstrated by the coach. No practical tethered swimming trials were performed during familiarisation, in order to avoid any acute kinematic carry-over that could confound the pre-intervention baseline measurement. Importantly, the experienced subgroup had acquired their exposure to tethered swimming during regular training in the weeks and months preceding the study, rather than acutely on the testing day; thus, neither subgroup experienced acute tethered-swimming carry-over into the baseline measurement. Consequently, the two subgroups (with vs. without prior tethered-swimming experience) differed in their cumulative practical exposure to the modality; this difference was not part of the primary aim and was examined post hoc as an exploratory factor. All procedures were coordinated by two experienced swimming coaches holding Level I coaching certification issued by the Polish Swimming Federation.

### Sample size estimation

An a priori power analysis was performed using G*Power for a within-subject comparison between two conditions. Assuming a two-tailed test, an alpha level of 0.05, a desired statistical power of 0.80, and a medium effect size (Cohen’s d_z_ = 0.50), the estimated required sample size was 34 participants.

### Study design

Recruitment and data collection were conducted between 14 June 2024 and 21 June 2024 at the swimming pool located at Koncertowa 4, 02–785 Warsaw, Poland.

**Fig. 1:**
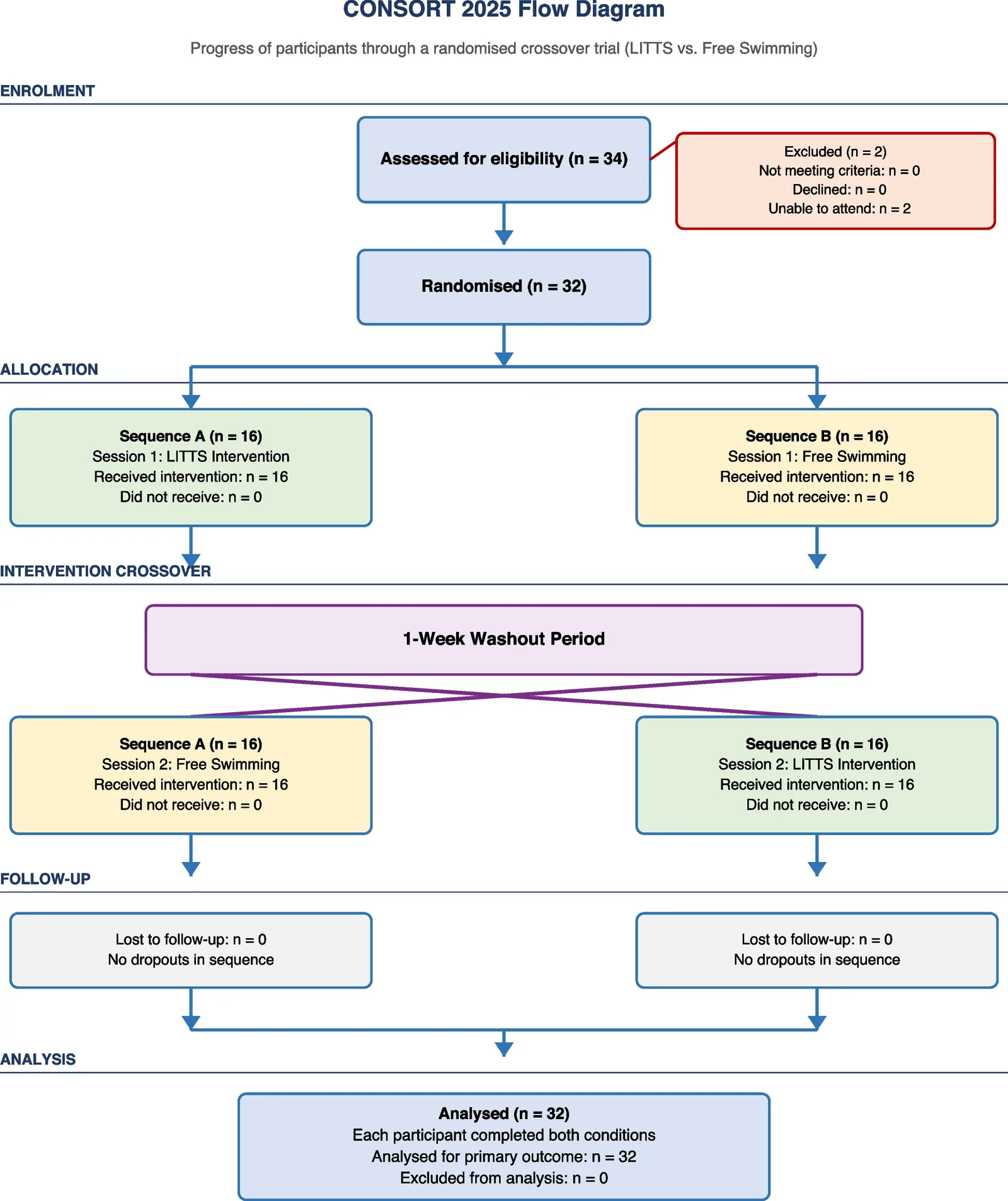
CONSORT 2025 flow diagram showing the progress of participants through the randomised crossover trial (LITTS vs. free-swimming condition). Of 34 swimmers assessed for eligibility, 2 were excluded (unable to attend) and 32 were randomised in a 1:1 ratio to one of two sequences. Sequence **A** performed the LITTS condition in Session 1 and the free-swimming condition in Session 2; Sequence **B** performed the conditions in the reverse order. The two sessions were separated by a one-week washout period. All 32 participants completed both conditions with no dropouts and were included in the analysis of the primary outcome. The diagram was prepared according to the CONSORT 2025 statement

This study employed a randomised crossover trial design. Of the 34 swimmers initially screened and deemed eligible for participation, 2 were unable to take part in the testing sessions due to an acute upper respiratory tract infection (common cold) and were therefore excluded from the study. The remaining 32 swimmers were randomly allocated in a 1:1 ratio to one of two intervention sequences (Sequence A and Sequence B). This trial was conducted and reported in accordance with the CONSORT 2025 guidelines, and a completed CONSORT checklist is provided as a supplementary file. The flow of participants through the trial is presented in Fig. 1 (CONSORT 2025 flow diagram).

The random allocation sequence was generated by MS using simple randomisation with the RAND() function in Microsoft Excel (Microsoft Corporation, Redmond, WA, USA): a random number was assigned to each participant and participants were sorted in ascending order of these values, with the first 16 allocated to Sequence A and the remaining 16 to Sequence B. MS, together with a certified swimming coach (Level I, Polish Swimming Federation), enrolled participants and assigned them to the intervention sequences according to the pre-generated allocation list.

During the 1-week washout period between testing sessions, all participants continued their regular swimming training programme as scheduled by their club coach. The training programme was identical for all participants, consisting of 7–8 sessions per week of standard club training. No additional tethered swimming training was performed during the washout period.

Performance trials were conducted individually, with one swimmer tested at a time.

### Procedure

Both groups performed the same warm-up, in accordance with the protocol proposed by Skorulski (2025):

Warm-up:

300 m free to choose.

12 × 25 m medley start in 40 s.

300 m crawl (very easy).

3 × 100 m start in 120 s (1 × 10 m crawl all-out/90 m very easy; 1 × progressive with increasing speed; 1 × breathing every 5 strokes).

Next: Group A additionally performed 6 × 10 cycles of low-intensity front crawl while tethered, with a 10-s break between sets. Group B performed 6 × 50 m of low-intensity front crawl, starting every 60 s. During the exercises, participants were instructed to focus on stroke length and correct body position in the water. Immediately upon task completion, accelerometer measurements were obtained. One week later, the procedure was repeated, with the final exercise crossed over between groups.

The two intervention protocols were matched for total duration (~ 7 min from the end of the warm-up to the start of the post-measurement trial) rather than for work volume. This was intentional: the time elapsed between the intervention and the performance measurement was considered the critical variable, given the time-dependent nature of priming effects. The additional time required for tethered setup and detachment in Group A was accounted for in the protocol design, resulting in comparable overall intervention windows across conditions.

Low-intensity was defined as exercise performed below the aerobic threshold, operationalised as heart rate < 150 bpm, consistent with the En1/A1 training zone described for competitive swimmers [27, 28]. Heart rate was monitored continuously in real time to account for interindividual variation in aerobic threshold (see Instruments section).

The measurements were conducted in a 25-m swimming pool. Participants were instructed to complete a 50-m front-crawl swim at maximal speed. Rather than starting from the blocks, the athletes began the trial by pushing off from the pool wall and completed the 50 m as two 25-m lengths with a tumble turn, consistent with standard front-crawl race execution.

All workouts were instructed and coordinated by experienced swimming coaches with Level I certification from the Polish National Swimming Association.

An elastic tether was used, which allowed unrestricted movement while maintaining the attachment point. This distinguishes the present protocol from rigid tethered systems typically used as measurement tools.

This study was designed and reported in accordance with the CONSORT 2025 guidelines, with extensions for randomised crossover trials. A completed CONSORT checklist is provided as an additional file (Fig. 1).

### Instruments

**Fig. 2:**
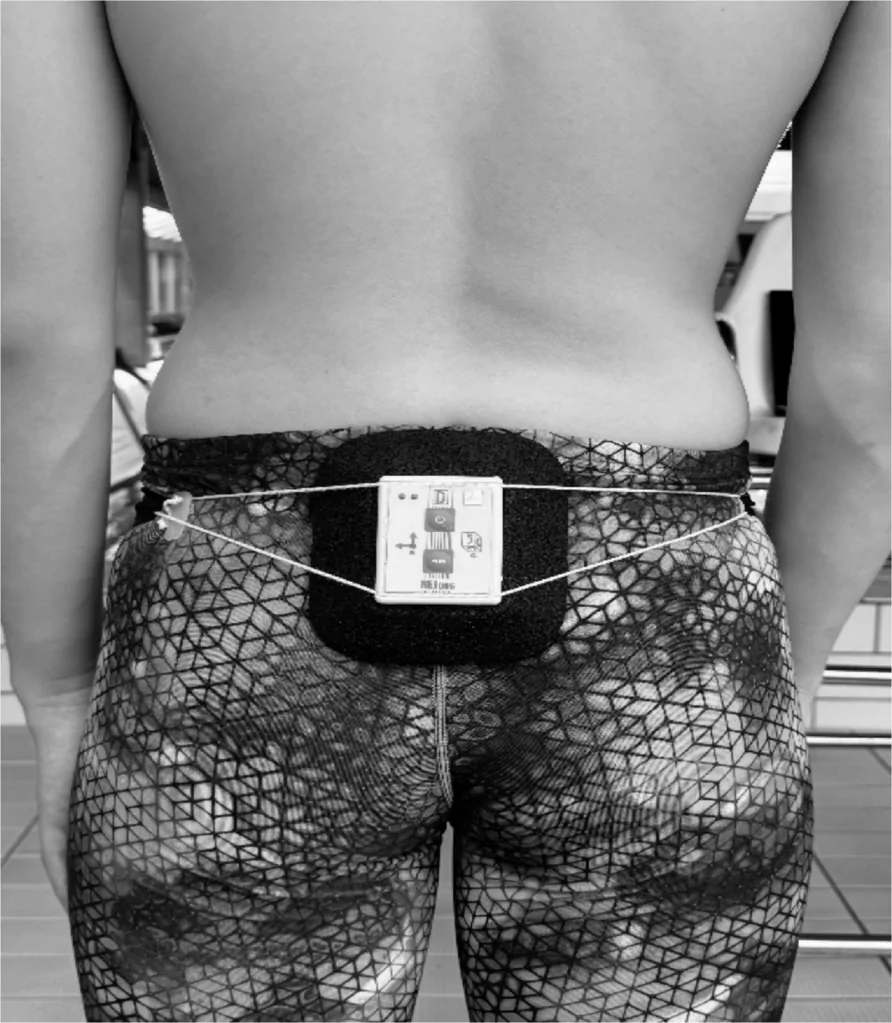
Method of fixing of the device for direct recording of triaxial accelerations and angular velocities of rotation

For each swimmer, individual kinematic features of the mean hip motion were evaluated separately for the first and second halves of the 50-m trial. Variations in velocity and acceleration were recorded using a data logger (REJ62g; JD Jarosław Doliński, Warsaw, Poland) incorporating a tri-axial gyroscope and a tri-axial accelerometer. The unit (65 × 50 × 30 mm; 95 g) was mounted on the lower back on the posterior aspect of the pelvic girdle. To minimise hydrodynamic drag and to stabilise the sensor, it was embedded in foam (Fig. 2). Fixation was achieved with a custom two-part belt: a non-elastic cord held the recorder firmly in place, while an elastic strap was wrapped around the lower abdomen.

Signals were sampled at 200 Hz. Linear acceleration was acquired within a range of ± 2 g and low-pass filtered (cut-off: 292 Hz). Angular velocity associated with rotational movements was measured with a range of ± 500 deg·s⁻^1^ and filtered using a low-pass filter with a 93 Hz cut-off. Both cut-off frequencies represent hardware-defined anti-aliasing filter parameters of the data logger and cannot be modified by the user.

Accelerometer accuracy was assessed under static conditions by comparison with gravitational acceleration, resulting in an absolute error of ± 0.2 m·s⁻^2^. Gyroscope performance was evaluated indirectly by measuring and computing the device rotation angle over a 90° excursion about each axis. The absolute error was ± 1° for the calculated angle and ± 0.6 deg·s⁻^1^ for angular velocity.

The recorded time series were subsequently smoothed with a fourth-order low-pass Butterworth filter (cut-off: 20 Hz). This cut-off frequency was selected to limit any reduction in the computed motion-velocity amplitude to no more than 0.5%, while preserving the key features of the acceleration waveform required for movement analysis.

Swimming cycles were identified from the body-roll signal. The roll angle was obtained by integrating the angular-velocity signal recorded by the gyroscope. Stroke boundaries were defined by the extrema of the roll-angle waveform, which correspond to zero-crossings of the angular-velocity signal: the onset of the right-side stroke was identified at the minimum roll angle following the preceding left-side stroke (where the angular velocity changed sign from negative to positive), and the onset of the left-side stroke at the maximum roll angle following the preceding right-side stroke (where the angular velocity changed sign from positive to negative). Left and right strokes were therefore segmented separately, consistent with the limb-specific (Left/Right) analysis.

Heart rate was monitored continuously during the intervention using Polar Verity Sense optical heart rate monitors (Polar Electro Oy, Kempele, Finland) worn on the forehead beneath the swim cap, with data displayed in real time on Polar Team application (iPad). Each participant was assigned a colour-coded indicator on the display; when heart rate exceeded 150 bpm, the indicator changed colour, prompting the participant to reduce exercise intensity. This visual biofeedback system ensured compliance with the low-intensity criterion throughout the intervention. This temple placement is the manufacturer-recommended position for swimming and differs from the arm placement; the validity of the device across these locations has been examined previously [29]. Because the sensor was used solely to confirm that the intervention remained at low intensity and did not contribute to any outcome variable, its placement is unlikely to have affected the study results.

### Outcome measure

Using the recorded signals, cycle-averaged numerical descriptors were derived for the swimming cycle, including arithmetic means and standard deviations of relative timing, all referenced to the onset of the cycle. All computations were performed with proprietary software STA1v0 (Zbigniew Staniak, Institute of Sport—National Research Institute, Poland).

The following variables relating to and describing individual body movements in three axes were measured and analysed:

*Body roll*

- ω_max_R [deg/s] – angular velocity around the vertical axis during rolling movements.
- A_max_R [deg] – maximum angle of pelvic tilt around the vertical axis during rotational movements.

*Yaw rotation*

- ω_max_Y [deg/s] – angular velocity around the sagittal axis during yaw movement.
- at_max_ [m*s–^2^] – acceleration along the transverse axis.

*Pitch rotation*

- ω_max_P [deg/s]—angular velocity around the transverse axis
- as_max_ [m*s–^2^]* –* acceleration along the sagittal axis

*Progressive movement*

- avmax [m*s–2] – acceleration along the vertical axis.

*Performance variables*

- Time to achieved 50 m [s]
- Stroke length [m]

**Fig. 3:**
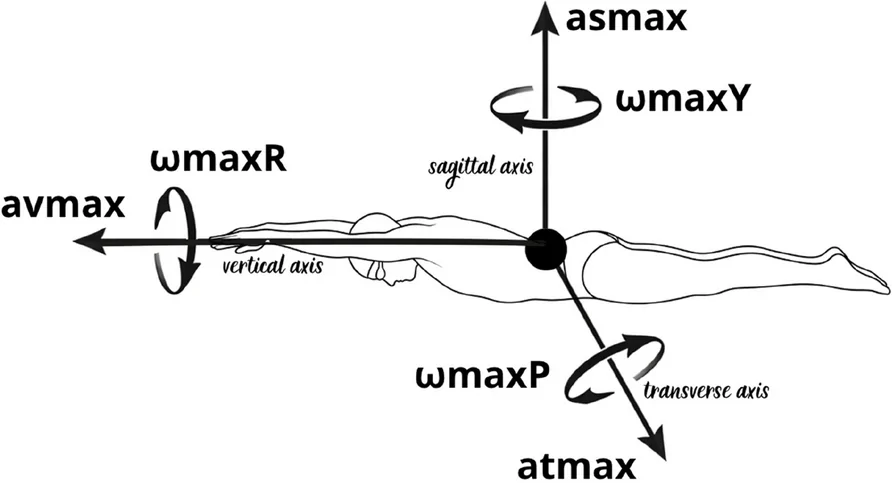
Layout and orientation of the measurement axes of triaxial accelerations and angular velocities of rotation. Notes: acceleration components: av_max_ – along the vertical axis, at_max_ –along the transverse axis, as_max_—acceleration along the sagittal axis and components of angular velocities of rotation: ω_max_R – around the vertical axis, ω_max_Y—around the sagittal axis, ω_max_P—around the transvers axis of swimmer’s body

A schematic representation of the analysed movement variables is presented in Fig. 3.

The data were analysed separately for movements involving the left upper limb (Left) and right upper limb (Right), as well as for the first lap of the swim (I25) and the second lap (II25).

To calculate the swimmers’ stroke length, above-water recordings were captured using a GoPro Hero 6 camera mounted on a poolside trolley, which followed the swimmer along the pool length. The start of the analysed section was defined as the stroke initiation point (SIP), i.e., the moment the first arm movement began after push-off from the wall – identified as the moment of initial arm separation from the streamlined position following push-off from the wall. From the SIP, the actual distance swum to the end of the pool, that is, until contact with the wall, was measured, and the total number of arm movements performed over this section was counted. Stroke length for each pool length was then calculated as the distance covered divided by the number of arm strokes. The same procedure was applied to the second length. Performance time was determined from video analysis (GoPro Hero 6, 60 fps; temporal resolution ≈ 0.017 s), measured from the moment of foot push-off from the wall to the moment of hand touch on the wall at the finish. This method was applied consistently across both testing conditions.

### Statistical analysis

Before analysis, the raw data were processed to include only complete swimming cycles. The streamline phase and turning were excluded from the kinematic analysis. Specifically, the arm separation from streamline and first pull following push-off, as well as the final pull before the turn, were excluded from each length in accordance with the analytical protocol applied in previous work with this device [20, 30], to ensure that only mid-pool steady-state cycles were included in the analysis.

Statistical analyses were performed using STATISTICA v. 13.1 software (TIBCO Software Inc., 2017). The normality of the data distribution was assessed using the Kolmogorov–Smirnov test, with a threshold of p-value > 0.05 used to confirm normality of a distribution. None of the analysed variables deviated from normality. The assumption of sphericity was met for all analysed variables, and homogeneity of variance was confirmed using Levene’s test.

A repeated-measures ANOVA (general linear model) was used, with three within-subject factors (STIMULUS: tethered vs free; LAP: first vs second; SIDE: left vs right) and one between-subject factor (GROUP: prior tethered experience vs no prior experience), the latter included as an exploratory term that did not form part of the primary aim.

The significance level was set at α = 0.05. Results were presented as means ± standard deviations, as well as 95% confidence intervals. Effect sizes were calculated using partial eta squared (ηp^2^) and interpreted as small (0.01), medium (0.06), and large (0.14), following thresholds commonly used in swimming-related research [31].

Because some of the analysed indicators described the same movement component (2 variables constituting a common ‘family’ for a single movement), Type I error was controlled within these families using the Holm–Bonferroni adjustment. No global correction was applied to all variables simultaneously, as individual indicators were treated as separate descriptors of movement performance. Table 1 shows both unadjusted (p_raw) and adjusted (p_HB) values for families comprising more than one variable.

**Table 1:** Acute effects of Low-Intensity Technical Tethered Swimming (LITTS) on performance and front crawl kinematic variables: descriptive statistics and repeated-measures ANOVA with Holm-Bonferroni correction

Family	Variables	Protocol		F	p	η^2^	p_HB
		**Free Swimming**	**Tethered Swimming**				
Time_50m	**Time_50m [s]**	30.97 ± 1.99 CI: 30.25—31.69	30.69 ± 1.91 CI: 30.00—31.38	9.71	0.004	0.24	-
Stroke	**Stroke_50m [m]**	0.92 ± 0.07 CI: 0.90—0.95	0.97 ± 0.09 CI: 0.94—1.00	47.09	0.001	0.61	-
Translational	**av**_**max**_ [m*s–^**2**^**]**	16.46 ± 2.28 CI: 15.64—17.28	14.04 ± 2.51 CI: 13.14—14.94	8.24	0.007	0.22	-
Yaw	**at**_**max**_ [m*s–^**2**^**]**	12.09 ± 3.08 CI: 10.98—13.20	12.07 ± 3.48 CI: 10.81—13.33	0.001	0.97	0.01	0.97
	**ω**_**max**_**Y [deg/s]**	109.5 ± 21.5 CI: 101.75—117.25	99.7 ± 25.2 CI: 90.61—108.79	10.85	0.003	0.27	0.006
Roll	**ω**_**max**_**R [deg/s]**	235.9 ± 50.1 CI: 217.83—253.97	238.5 ± 52.1 CI: 219.71—257.29	0.31	0.586	0.01	0.586
	**A**_**max**_**R [deg]**	29.72 ± 4.56 CI: 28.08—31.37	31.33 ± 4.61 CI: 29.67—32.99	6.19	0.021	0.17	0.042
Pitch	**as**_**max**_ [m*s–^**2**^**]**	3.95 ± 1.42 CI: 3.44—4.46	3.52 ± 1.08 CI: 3.13—3.91	11.23	0.015	0.19	0.030
	**ω**_**max**_**P** **[deg/s]**	53.81 ± 14.96 CI: 48.42 −59.20	48.58 ± 18.31 CI: 41.98 −55.18	5.43	0.026	0.16	0.030

Values are reported as mean ± SD for Low-Intensity Technical Tethered Swimming (LITTS) stimulus and the corresponding free-swimming technical conditions. For each outcome, the table provides the F statistic, p-value (p), and effect size (η^2^; partial eta squared) from repeated-measures ANOVA for the acute change (free swimming vs tether swimming). To control for multiple comparisons within predefined families of conceptually related variables (Performance, Stroke, Translational, Yaw, Roll, Pitch), p values were adjusted using the Holm–Bonferroni procedure (p_HB); for single-variable families, p_HB equals p. Statistical significance was set at α = 0.05. Abbreviations: ω_max_R-maximum roll angular velocity; ω_max_Y-maximum yaw angular velocity; A_max_R-maximum roll angle; atmax—acceleration along the transverse axis; ω_max_P—angular velocity around the transverse axis; as_max_—acceleration along the sagittal axis; av_max_—acceleration along the vertical axis; Time_50m—time to swim 50 m front-crawl; Stroke_50m—stroke length over a distance of 50 m using the front- crawl stroke

Correction for multiple comparisons was not applied across all kinematic outcomes, as each variable captures a distinct aspect of movement execution. However, to control the family-wise error rate within each movement component, we applied a Holm–Bonferroni adjustment to sets of two kinematic variables describing the same movement (within-component comparisons).

To characterise individual responses, the proportion of participants whose change occurred in the direction of the group-level effect was computed for each variable presented in the individual-response figure.

## Results

The results are presented as mean values for the analysed kinematic variables, including velocity, acceleration, stroke length, and time taken to cover the prescribed distance. Table 1 presents the means ± SD, p-values, F statistics, η^2^, and Holm–Bonferroni correction (p_HB) for the analysed variables. Significant group-level changes in kinematic parameters were observed following the Low-Intensity Technical Tethered Swimming intervention, as supported by the reported significance levels and effect sizes; the consistency of these responses across participants is examined in the Individual Responses section below.

### 50-m performance time

The analysis revealed a significant difference (F(1,29) = 9.71; *p* = 0.005; η^2^ = 0.24) in 50-m performance time (Time_50m) in the two testing conditions. Time_50m was significantly shorter (~ 1%) after LITTS (30.69 ± 1.91 s) compared with the free-swimming condition (30.97 ± 1.99 s).

### Stroke length

The analysis revealed significant differences (F(1,29) = 47.09; *p* = 0.001; η^2^ = 0.61) in stroke length following the LITTS intervention.

### Translational motion

The study showed a significant group-level decrease (~ 15%) in acceleration along the vertical axis after the application of LITTS (F(1,29) = 8.24; *p* = 0.007; η^2^ = 0.22), although this response varied across participants (see Individual Responses).

### Body roll

Body-roll analysis revealed a statistically significant 5% increase in the maximum angle of pelvic tilt around the vertical axis (AmaxR) during LITTS compared with free swimming (F(1,29) = 6.19, *p* = 0.020, η^2^ = 0.17).

These differences remained statistically significant after applying the Holm–Bonferroni correction (p_HB = 0.042). No statistically significant differences were observed in maximum angular velocity around the vertical axis (ωmaxR). No significant main effect of sex or sex × time/stimulus interaction was found, indicating that the response to the applied stimulus was comparable between females and males.

No significant group × tether/no-tether interaction effect was observed, indicating that the responses in body-roll variables were similar regardless of prior tethered-swimming experience.

### Yaw rotation

The analysis revealed a significant difference in maximum angular velocity about the sagittal axis (ω_max_Y) between the two testing conditions. ω_max_Y following the LITTS condition showed a marked decrease compared with the free-swimming condition. The results remained significant after Holm–Bonferroni correction (p_HB = 0.006). No significant differences were observed in maximum acceleration along the transverse axis (at_max_).

No significant group × tether/no-tether interaction was detected, indicating that the responses in yaw-rotation variables were similar regardless of prior tethered-swimming experience.

### Pitch rotation

Analysis of pitch movement parameters between free swimming and the LITTS stimulus showed significant differences (F(1,29) = 11.27, *p* = 0.015, η^2^ = 0.19) in maximum acceleration along the sagittal axis (as_max_) and significant differences (F(1,29) = 5.43, *p* = 0.026, η^2^ = 0.16) in maximum angular velocity around the transverse axis (ω_max_P).

These differences remained statistically significant after applying the Holm–Bonferroni correction (as_max_/ω_max_P; p_HB = 0.030).

Following LITTS, decreases in as_max_ (by ~ 11%) and ω_max_P (by ~ 10%) were observed. No significant group × tether/no-tether interaction effect was observed, indicating that the responses in pitch rotation variables were similar regardless of prior tethered-swimming experience.

**Fig. 4:**
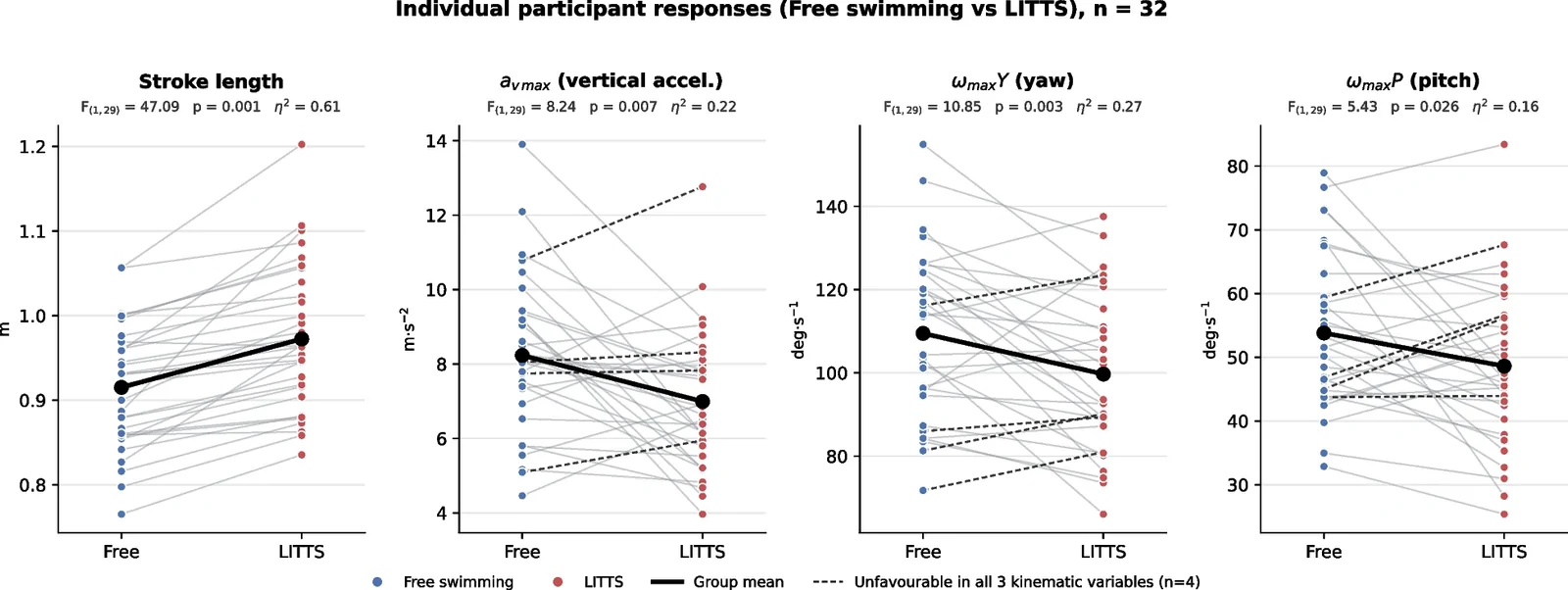
Individual participant responses to the Low-Intensity Technical Tethered Swimming (LITTS) condition relative to the free-swimming condition (n = 32). Each thin grey line represents one participant; blue dots denote the free-swimming condition and red dots the LITTS condition, and the bold black line indicates the group mean. Panels show stroke length, vertical-axis acceleration (avmax), maximum yaw angular velocity (ωmaxY) and maximum pitch angular velocity (ωmaxP). Above each panel, the F statistic, p-value and effect size (η², partial eta squared) from the repeated-measures ANOVA are reported. Dashed lines identify the four participants whose response was unfavourable across all three kinematic variables (avmax, ωmaxY, ωmaxP); in each of these four, stroke length nonetheless increased

Individual participant responses are presented in Fig. 4. The increase in stroke length following LITTS was highly consistent across the sample, with 31 of 32 participants (97%) improving (d_z_ = 1.21). Responses in the kinematic variables were more heterogeneous: a favourable (reduced) response was observed in 22 of 32 participants for aᵥ max (69%), 21 of 32 for ω max Y (66%) and 20 of 32 for ω max P (62%). Fourteen participants (44%) responded favourably in all three of these variables, whereas four (12.5%) showed an unfavourable change in all three; in each of these four, stroke length nonetheless increased.

## Discussion

The main finding of this study is that the LITTS protocol induced acute changes in swimming kinematics (hereafter referred to as acute technical refinement; ATR), characterised by a pattern of improvement in selected kinematic parameters. However, the improvement in 50 m front-crawl time was relatively small (~ 1%), corresponding to a 0.3 s reduction in swimming time. In competitive swimming, where outcomes are often decided by hundredths of a second, such an improvement has practical significance (for instance, the 0.08 s gap separating 3rd and 8th place in the men’s 50 m freestyle at the Paris 2024 Olympics). A similar performance enhancement was reported by Lee et al. [24], who reduced the mean 50 m breaststroke time by approximately 0.35 s (~ 1%) compared with the standard warm-up.

In addition to the significant improvement, a 6% increase in swimming stroke length was observed. Notably, this kinematic effect was characterised by a large effect size (η2 = 0.61), indicating a strong intervention response. Previous literature emphasises stroke length as one of the most important factors influencing swimming performance. Studies indicate that faster swimmers cover greater distances with each stroke rather than simply increasing their stroke frequency [32–34]. In order to explain, at least partially, the biomechanical mechanisms underlying this significant increase in stroke length, our research indicates changes in motion kinematics as an effect of LITTS, described in three main areas: body roll, rotation around the transverse axis, and pitch.

### Body roll

Accelerometric analysis of rotational movements about the body’s vertical axis uses variables that describe both the amplitude of rotation, represented by the maximum pelvic tilt angle, and its dynamics, assessed through the analysis of angular velocity [35–37]. An approximately 5% increase in the maximum rotation angle (A_max_R) was observed while maximum angular velocity about the vertical axis remained constant. This can be interpreted as a technical adjustment: owing to LITTS, swimmers increased the range of pelvic rotation while maintaining a similar maximum angular velocity. According to Yanai’s conclusions, such a strategy (i.e. increasing A_max_R without increasing ω_max_R) may be beneficial if it results in greater pulling efficiency and low energy cost [38]. The results of the body-roll analysis suggest that LITTS may influence rotational movements about the vertical axis.

### Yaw rotation

Intensification of yaw rotation movements is a common phenomenon in swimmers, particularly associated with asymmetrical arm movements, the breathing phase [39], and fatigue [30]. Studies have shown that swimmers should maximise forward displacement while minimising yaw movements in order to achieve the most balanced body position possible. In this study, the observed reduction in angular velocity indicates that the athletes performed less rapid tilting movements. A decrease in ω_max_​Y is typically associated with a smoother trajectory (reduced side-to-side wobbling). Researchers emphasise that intensifying yaw movements can increase drag by disrupting the linearity of movement through the water [30, 39–41].

### Pitch movements

In this study, pitch movements are described using two variables: maximum acceleration in the sagittal axis direction (as_max_) and maximum angular velocity about the transverse axis (ω_max_P). This movement manifests as a rise and fall of the pelvis. From a hydrodynamic perspective, any deviation from the body’s axial position substantially increases water resistance [42]. This is supported by previous research, which identifies this movement as an important indicator of swimming technique and economy [43].

Recent literature indicates that pitch movements intensify as a result of inadequate leg and arm actions, which are strongly associated with fatigue, particularly over longer distances [44, 45]. Washino et al. [45] observed that the propulsive phases of the arms had a greater effect on the increase in pitch movements than the non-propulsive phases. Given its proposed tendency to induce hydrodynamic instability, LITTS may influence arm-movement kinematics, with potential effects extending well beyond biomechanics alone. LITTS may function as a constraints-led exercise that amplifies hydrodynamic errors, thereby providing a movement-correcting stimulus consistent with the observed pattern of kinematic changes (ATR). In contrast, the present study investigates a technically oriented form of priming that operates independently of high-intensity activation.

### Intra-cycle velocity variation in swimming

Following LITTS, a significant decrease (~ 15%) in maximum vertical-axis acceleration (avmax) was observed. Given that any change in instantaneous swimming velocity is preceded by a change in acceleration, avmax may serve as an indirect indicator of intra-cycle velocity variation (IVV), the continuous fluctuation of swimming speed within a single stroke cycle. In the literature, IVV is considered a negative phenomenon, as the work required for re-acceleration after a deceleration phase substantially exceeds that needed to maintain constant velocity [46, 47].

Elite swimmers have been shown to produce lower peak acceleration values per cycle than amateur swimmers while maintaining higher overall speed, which directly reflects lower IVV [48].

The observed reduction in avmax may therefore suggest a more stable intra-cycle velocity profile following LITTS, although IVV was not directly measured in the present study, and this interpretation should be treated with caution.

### Alternative explanations

Although the hypothesis emphasises the role of priming in adjusting kinematics, another neurophysiological phenomenon may also provide a compelling explanation for the observed changes. Kinaesthetic after-effect is a phenomenon in which the observed effect results from prior activity performed under altered conditions (e.g. greater resistance or a heavier implement) [49]. After being released from the tether, the swimmer experiences a sense of lightness, similar to that reported by Nakamoto et al. [49], in which a baseball player who initially swung a heavier bat experienced lightness after switching to a lighter one. This occurs through perceptual distortion of the sense of limb position, the intensity of muscle contraction, or tool resistance immediately after completing work with the preceding (changed) stimulus. This effect is thought to arise from a transient perceptual distortion in the sense of limb position, muscular effort, or external resistance immediately following the removal of the preceding altered stimulus.

### Individual response patterns

Inspection of individual responses (Fig. 4) indicated that the improvement in stroke length was near-universal, whereas the accompanying kinematic adjustments followed more individualised patterns. This dissociation is consistent with the constraints-led interpretation advanced here: rather than imposing a single uniform movement solution, LITTS may act as a task constraint within which each swimmer self-organises towards an individually effective coordination pattern, with stroke length representing the convergent functional outcome. Notably, the four participants in whom all three displayed kinematic variables (av_max_, ω_max_Y, ω_max_P) changed in an unfavourable direction were all swimmers who had previously trained with tethered swimming. Although based on a very small subgroup (*n* = 4) and unsupported by a statistically significant group effect, this observation may tentatively indicate that prior familiarity with the tethered condition attenuates the acute response, consistent with the proposed error-augmentation mechanism: the constraint may elicit a weaker corrective signal when the task is no longer novel to the swimmer. This possibility warrants examination in adequately powered future studies. Importantly, stroke length increased in all four of these participants.

### Limitations

Although formal test–retest reliability (typical error or intraclass correlation) was not quantified for the outcome measures in the present sample, several considerations mitigate this. Performance time was derived from video analysis at 60 fps (temporal resolution ≈ 0.017 s) using an identical procedure across both conditions, so the ~ 0.3 s within-participant difference is well above the measurement resolution and unlikely to reflect timing error. For the kinematic variables, the device's static measurement error (± 0.2 m·s⁻^2^; ± 1°; ± 0.6 deg·s⁻^1^) was small relative to most of the observed changes, although the smallest change (roll angle, ~ 1.6°) approached this resolution and should be interpreted with corresponding caution.

The interpretation is further supported by earlier reports on this device, which have demonstrated its compatibility with video analysis for identifying swimming cycles, its sensitivity to changes in movement phase times, and its ability to detect changes in front crawl technique under the influence of fatigue and muscle tremor [20, 30, 50–52]. At the same time, the generalisability of the results to adult athletes should be considered limited, as a more mature group of athletes may exhibit greater stability in their movement patterns. Given the relatively young sample, natural performance variability and motivational responses to the testing procedure cannot be fully excluded as contributing factors. It should also be acknowledged that participants without prior experience received only verbal and observational familiarisation rather than full practical familiarisation, which has been recommended before formal tethered-swimming evaluation [53]; some heterogeneity in baseline familiarity with the modality may therefore have remained. However, the consistency of the observed effects across both participants familiar with tethered swimming and those encountering it for the first time provides some support for a genuine intervention response rather than a novelty effect. More broadly, although the two conditions were matched for total duration rather than exercise volume (see Methods), they differed in aspects beyond the tether itself, including exercise volume and the structure of the preparatory task. The acute changes observed after LITTS therefore cannot be attributed exclusively to the tethering constraint, and the contribution of these other differences cannot be fully isolated within the present design.

### Perspective section

Based on the present findings, LITTS may be a useful addition to pre-competition or pre-training preparation, particularly when placed after the warm-up and before sprint sets [54]. Given the low-intensity nature of the protocol and the absence of peripheral fatigue, it may represent a technically oriented alternative to traditional high-intensity priming methods. Coaches working with young competitive swimmers may consider incorporating LITTS as a preparatory tool. The precise time window during which the effect persists was not assessed in the present study and is identified as a priority for future research.

### Prior experience

Because the sample included swimmers with and without prior tethered-swimming experience, we examined prior experience as an exploratory factor, to test whether the acute effect was driven by familiarity with the modality rather than by the intervention itself. No evidence was found that prior exposure to this type of exercise significantly influenced the magnitude of the observed effect. Statistical analysis revealed no significant differences between the groups after the application of LITTS. Both participants undertaking LITTS for the first time and those already familiar with it achieved comparable positive effects. This suggests that the response to the applied stimulus was independent of previous experience in performing this type of exercise.

While these findings tentatively suggest that the response to LITTS may not depend strongly on prior tethered swimming experience, this interpretation should be treated with caution given the relatively small sample size (*n* = 32), which limits the statistical power to detect subgroup differences. The absence of a significant group × condition interaction does not preclude the possibility that learning or habituation effects contributed to the observed changes.

### Future research directions

Gaps in current knowledge provide a basis for designing future research, which should address the following issues: (1) precisely determining the time window of the effect induced by LITTS; (2) describing the impact of LITTS on other swimming styles; and (3) investigating whether neurodevelopmental maturity, chronological age, and training experience influence susceptibility to the acute effects induced by LITTS.

## Conclusions

The aim of this study was to investigate the acute effects of LITTS as a technical priming strategy. The findings provide preliminary evidence of acute kinematic changes (ATR) following LITTS, characterised by improvements in selected kinematic variables that were not accompanied by increases in physiological load.

The low-intensity nature of the LITTS protocol suggests that the observed kinematic changes are unlikely to be attributable to PAP or PAPE mechanisms, which typically require high-intensity activation and involve peripheral fatigue [12, 13]. Rather, the findings are consistent with a technically oriented priming effect, though the precise neuromotor mechanisms remain to be established in future research [11]. Further research is required to clarify the mechanisms underlying these responses and to determine whether LITTS represents a distinct form of technical priming.

## Data Availability

The anonymized processed dataset supporting the conclusions of this article is available in the Zenodo repository, access via: https://doi.org/10.5281/zenodo.19651175 (Data are available at Zenodo: https://zenodo.org/records/19651175).

## Abbreviations

ANOVA: Analysis of variance
ATR: Acute technical refinement
CLA: Constraints-led approach
CONSORT: Consolidated Standards of Reporting Trials
IVV: Intra-cycle velocity variation
LITTS: Low-intensity technical tethered swimming
PAP: Post-activation potentiation
PAPE: Post-activation performance enhancement
SD: Standard deviation
SIP: Stroke initiation point
TTS: Technical tethered swimming
ωmaxR: Maximum angular velocity around the vertical axis (body roll)
A_max_R: Maximum pelvic tilt angle around the vertical axis
ω_max_Y: Maximum angular velocity around the sagittal axis (yaw)
at_max_: Maximum acceleration along the transverse axis
ωmaxP: Maximum angular velocity around the transverse axis (pitch)
as_max_: Maximum acceleration along the sagittal axis
av_max_: Maximum acceleration along the vertical axis
